# Cellular heterogeneity in the 16HBE14o ^−^ airway epithelial line impacts biological readouts

**DOI:** 10.14814/phy2.15700

**Published:** 2023-06-03

**Authors:** Jenny L. Kerschner, Alekh Paranjapye, Ann Harris

**Affiliations:** ^1^ Department of Genetics and Genome Sciences Case Western Reserve University Cleveland Ohio USA; ^2^ Present address: Department of Genetics University of Pennsylvania Philadelphia Pennsylvania USA

## Abstract

The airway epithelial cell line, 16HBE14o^−^, is an important cell model for studying airway disease. 16HBE14o^−^ cells were originally generated from primary human bronchial epithelial cells by SV40‐mediated immortalization, a process that is associated with genomic instability through long‐term culture. Here, we explore the heterogeneity of these cells, with respect to expression of the cystic fibrosis transmembrane conductance regulator (CFTR) transcript and protein. We isolate clones of 16HBE14o^−^ with stably higher and lower levels of CFTR in comparison to bulk 16HBE14o^−^, designated CFTR^high^ and CFTR^low^. Detailed characterization of the *CFTR* locus in these clones by ATAC‐seq and 4C‐seq showed open chromatin profiles and higher order chromatin structure that correlate with *CFTR* expression levels. Transcriptomic profiling of CFTR^high^ and CFTR^low^ cells showed that the CFTR^high^ cells had an elevated inflammatory/innate immune response phenotype. These results encourage caution in interpreting functional data from clonal lines of 16HBE14o^−^ cells, generated after genomic or other manipulations.

## 1 INTRODUCTION

Advances in understanding human lung disease are largely dependent on the availability of robust in vitro cell culture models. Although primary airway epithelial cell cultures are preferred, their availability is always limited, so many alternative culture models were established. These include diverse lung cancer cell lines of different cellular origins, immortalized cell lines, and more recently induced pluripotent stem cell (iPSC)‐derived lung cell types. The choice of model system is largely tailored to the inherent properties of the cell line and their relevance to the topic of investigation. In the context of developing gene‐editing therapeutics, the ability to perform genomic manipulations with CRISPR/Cas9 and subsequently isolate single cell‐derived populations with extended lifespan is critical. Among cell lines frequently used for these studies are airway cells immortalized with simian virus 40 (SV40) or derivative plasmids such as origin‐defective SV40 (Fromm & Berg, 1982). Immortalization results from the stable random integration of one to a few copies of the SV40 genome and is dependent on continued expression the SV40 large T antigen (reviewed in [Pipas, 2009]). Among such immortalized airway epithelial cell lines that are routinely used in airway disease research are BEAS‐2B (adeno‐SV40 hybrid) (Reddel et al., 1988) and 16HBE14o^−^ (SV40 ori‐ plasmid) (Cozens et al., 1994). Disease‐specific airway epithelial cell lines include the cystic fibrosis (CF) bronchial cells IB3‐1 (adeno‐SV40 hybrid) (Zeitlin et al., 1991) and ΣCFNPE14o^−^ (SV40 ori‐ plasmid) nasal epithelial cells (Kunzelmann et al., 1995).

CF is a life‐limiting autosomal recessive disease, caused by mutations in the cystic fibrosis transmembrane conductance regulator (*CFTR*) gene. Loss or aberrant function of the CFTR protein has a profound impact on multiple epithelial cell types in the airway, pancreas, intestines and male genital ducts, among other sites. In the airway, defective anion transport by mutant CFTR is associated with thickened mucus and a lung environment susceptible to persistent infection and inflammation. The 16HBE14o^−^ cell line which has maintained a cobblestone‐like epithelial morphology and expresses abundant *CFTR* transcripts and lower, but readily detectable levels of CFTR protein, is a valuable resource for CF research. When polarized on permeable supports these cells can form tight junctions and thus serve as a useful model for the study of barrier function and ion transport in respiratory disease (Callaghan et al., 2020; Heijink et al., 2012; Leir et al., 2003; Wan et al., 2000). They have also been used to investigate many aspects of airway epithelial biology including drug transport (Forbes, 2000), toxicology (Feng et al., 2015) and air pollution (Zhou et al., 2015).

In previous work, we used the 16HBE14o^−^ line, among others, to study the regulatory mechanisms governing *CFTR* expression in the airway epithelium. This line has the advantage over some others that it is amenable to genome editing and the derivation of single cell clones (Kerschner et al., 2022; NandyMazumdar et al., 2020; Paranjapye et al., 2022; Santos et al., 2023; Valley et al., 2019). We identified and characterized two strong airway‐selective enhancers at −35 kb and −44 kb upstream of the *CFTR* promoter (Kerschner et al., 2022; NandyMazumdar et al., 2020; NandyMazumdar et al., 2021; Paranjapye et al., 2022; Zhang et al., 2012, 2013, 2015). These enhancers, and many of the transcription factors and mechanisms driving their activity, are conserved in other airway cells including the lung carcinoma cell line Calu3, primary human bronchial epithelial cells, iPSC‐derived airway epithelial cells (Kerschner et al., 2021; Mutolo et al., 2018; NandyMazumdar et al., 2020, 2021; Paranjapye et al., 2022) and adult and fetal human tissues (Bergougnoux et al., 2014). DNA sequence analysis of 16HBE14o^−^ cells indicates that at least one copy of the pSV40 (ori‐) plasmid has integrated into intron 6 of the *CFTR* locus, and it has been suggested that this results in mono‐allelic expression of *CFTR* in these cells (Valley et al., 2019). Moreover, we observed substantial recruitment of RNA polymerase II (RNAPII) to the integration site (NandyMazumdar et al., 2020), though it is unclear whether this only occurs on the SV40 integrated allele.

In the context of isolating clonal wild‐type control lines for CRISPR/Cas9 genomic manipulations in 16HBE14o^−^ cells, we observed a wide variation in *CFTR* expression levels. This variation might result from off‐target gene editing or because different cells within the 16HBE14o^−^ population had variable endogenous *CFTR* expression level. We investigated the latter possibility by isolating clonal cell lines from a non‐edited population culture of 16HBE14o^−^ cells. We identified clones with reproducibly high or low levels of *CFTR* in relation to bulk 16HBE14o^−^. Here, we characterize these two populations of cells to determine their different identities, using genome‐wide transcriptional profiling and detailed analysis of the chromatin structure and 3D interactions at the *CFTR* locus.

## 2 MATERIALS AND METHODS

### 2.1 *CFTR* nomenclature

For consistency with our previous work (Gosalia & Harris, 2015), *CFTR* introns and exons are numbered using legacy nomenclature (Tsui & Dorfman, 2013). RefSeq conversion is published elsewhere (Yin et al., 2022).

### 2.2 Cell culture

Human bronchial epithelial 16HBE14o^−^ (Cozens et al., 1994) cell lines were cultured in Dulbecco's modified Eagle's medium, low glucose supplemented with 10% fetal bovine serum. Wild‐type 16HBE14o− (RRID:CVCL_0112), of male origin, were originally a gift from Dr. D.C. Gruenert. For RNA and protein isolation, approximately 200,000 cells were seeded into individual wells of a 12‐well plate, and media were replaced at 48 h. Following two PBS washes, cells were collected at 95%–100% confluence (~96 h post plating) for protein or RNA (see below).

### 2.3 Subcloning of 16HBE14o ^−^

16HBE4o^−^ were subcloned by manual single‐cell dilution and plated into 96‐well plates. Forty‐eight clones grown from a single cell were subcultured into 48‐well plates, with 12 clones further subcultured for profiling from 12‐well plates. RNA was collected at passages 7 and 9 after subcloning (p0), and lysates were collected at passages 8 and 10 post subcloning.

### 2.4 RNA preparation and reverse transcription quantitative PCR

Following the manufacturer's protocol, total RNA was extracted using TRIzol (Thermo Fisher, 15596018) and cDNA prepared using Taqman Reverse Transcription Reagents with random hexamers (Thermo Fisher, N8080234). Using primers listed in Table S1, qPCR performed with Taqman Fast Advanced Master Mix (Thermo Fisher, 4444557).

### 2.5 Western blot analysis

Cells were lysed in NET buffer (10 mM Tris–HCl, pH 7.5, 150 mM NaCl, 5 mM EDTA, 1% [v/v] Triton X‐100, 1X Protease Inhibitor Cocktail [Sigma‐Aldrich, P8430]). CFTR protein was extracted and western blots performed as described previously (Cai et al., 2015). Briefly, lysates were resolved on 4%/7% gels at 60–80 V using standard SDS‐PAGE protocols. Proteins were transferred to Immobilon‐P PVDF membranes (Millipore‐Sigma, IPVH00010) at 24 V overnight at 4°C. Membranes were probed with antibodies specific for CFTR (Cystic Fibrosis Foundation, CFF‐596, lot # TJ20200121100285, RRID:AB_2923486), β‐tubulin (Sigma‐Aldrich, T4026, lot # 128M4790V, RRID:AB_477577) and anti‐mouse‐HRP (Agilent/Dako, P0447, lot # 20051789, RRID:AB_2617137), and proteins detected with ECL Western Blotting Substrate (Pierce).

### 2.6 Omni assay for transposase accessible chromatin and deep sequencing

Omni assay for transposase accessible chromatin and deep sequencing (Omni‐ATAC‐seq) was performed on 50,000 cells as described previously (Corces et al., 2017) with minor modifications (NandyMazumdar et al., 2020). Library size distributions were visualized by TapeStation (Agilent) and quantified using the KAPA Library Quantification Kit (Roche). Libraries were pooled and sequenced on a NextSeq 550 at high output (Illumina) using 75 bp single reads. Data were processed by the ENCODE‐DCC/atac‐seq‐pipeline.

### 2.7 Chromosome conformation capture 4C‐seq

4C‐seq libraries were generated from 1 × 10^7^ cells as described previously (Krijger et al., 2020). Cross‐linked chromatin from 1 × 10^7^ cells was digested using NlaIII and DpnII as the primary or secondary restriction enzymes, respectively. 4C experiments were performed at least twice from independent passages. Viewpoint primer sequences and enzyme pairs are shown in Table S1. Quantification of 4C‐seq reads was generated using the pipe4C pipeline v1.1 (Krijger et al., 2020) with default parameters. Read density tracks of replicates were merged and then subtracted from bulk 16HBE14o^−^ using the deepTools bigwigCompare tools (Ramírez et al., 2016).

### 2.8 RNA‐seq

RNA was isolated from triplicate samples of clonal lines or bulk populations of 16HBE14o^−^ cells using TRIzol (Life Technologies) as described previously  (Dobin et al., 2013) and RNA‐seq was performed by standard protocols (SR 50 bp) on a NovaSeq 6000 following generation of random hexamer primed cDNA libraries using TruSeq Stranded mRNA Sample Preparation Kits (Illumina). Raw reads were aligned with STAR 2.7 (https://github.com/alexdobin/STAR, RRID:SCR_004463) (Dobin et al., 2013). Aligned reads were then assigned to genomic features with featureCounts version 1.6.3 (RRID:SCR_012919) in the Subread package (http://subread.sourceforge.net/, RRID:SCR_009803) (Liao et al., 2014) and differential gene expression was analyzed using DEseq2 version 1.22.1. (https://www.bioconductor.org/packages/release/bioc/html/DESeq2.html, RRID:SCR_015687) (Love et al., 2014).

### 2.9 Gene ontology

Differentially expressed genes were filtered using a basemean >30, fold change ≥+1.5 and ≤−1.5 and a Benjamini–Hochberg adjusted *p*‐value ≤0.01, and were read into DAVID 2021 to generate GO terms (Sherman et al., 2022).

## 3 RESULTS

### 3.1 The 16HBE14o ^−^ cell line contains subpopulations of cells with differing CFTR levels

**FIGURE 1:**
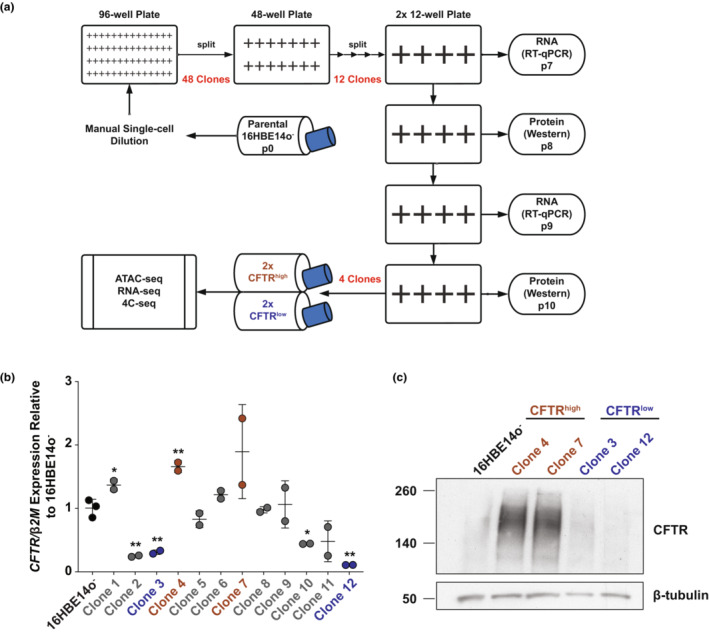
16HBE14o^−^ cell line contains two subpopulations expressing differing amounts of CFTR transcript and protein. (a) Schema illustrates the generation of 16HBE14o^−^ clones from single cells following manual dilution and subsequent analyses. (b) *CFTR* expression normalized to β*2M*, shown relative to 16HBE14o^−^ WT cells (*n* = 2). Cells at two non‐sequential passages were evaluated for 12 clones, with the four identified for subsequent analyses shown in brown (CFTR^high^) or blue (CFTR^low^). ** denotes *p* < 0.01 and * denotes *p* < 0.05, compared to 16HBE14o^−^ using unpaired *t*‐tests with Welch's correction. (c) Western blot showing CFTR protein levels with an antibody specific for CFTR in 16HBE14o^−^ cells and two clones each of CFTR^high^ and CFTR^low^ cells. β‐tubulin provides the loading control. The data shown are representative of the two separate passages surveyed.

The 16HBE14o^−^ airway epithelial line was manually diluted and expanded to generate clones of single‐cell origin, of which 12 were assayed for *CFTR* gene and CFTR protein expression (Figures 1 and S1). We identified clones in which CFTR levels were higher and lower than that of the bulk 16HBE14o^−^ WT cell population (Figures 1b,c and S1). Two clonal lines each with high *CFTR* transcript and protein expression (clones 4 and 7) and low CFTR (clones 3 and 12) were studied further and are referred to herein as CFTR^high^ and CFTR^low^. CFTR^high^ cells had a 1.7‐fold to 1.9‐fold increase in *CFTR* transcript and a substantial increase in CFTR protein compared to WT (Figures 1b,c and S1). CFTR^low^ cells showed a 3.1‐fold to 9.1‐fold decrease in *CFTR* transcript compared to WT and slightly reduced levels of CFTR protein (Figures 1b,c and S1). The *CFTR* transcript levels measured by reverse transcription quantitative PCR were confirmed in RNA‐seq data (see below) and were maintained over multiple passages, with passages 7 and 9 post‐subcloning assayed. These data show that within the 16HBE14o^−^ airway epithelial cell line, there exist subpopulations of cells expressing differing amounts of CFTR.

### 3.2 *CFTR* locus higher order chromatin structure and 3D organization differ in 16HBE14o ^−^ clonal lines and correlate with *CFTR* expression levels

*CFTR* expression is controlled by distal and intronic *cis*‐regulatory elements (CREs) and adopts cell‐type specific 3D conformations, which are required for normal CRE‐promoter interactions. We showed previously that deletion of CREs or depletion of activating transcription factors (TFs) disrupts these higher order chromatin interactions and alters *CFTR* expression levels (Kerschner et al., 2021; NandyMazumdar et al., 2020, 2021; Paranjapye et al., 2021, 2022; Yang et al., 2016; Yin et al., 2022). Hence, we next investigated whether the *CFTR* locus adopts different chromatin conformations and 3D interactions in the CFTR^high^ and CFTR^low^ clonal cells.

**FIGURE 2:**
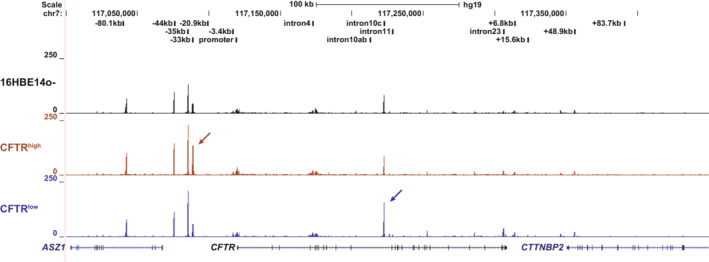
CFTR^high^ and CFTR^low^ clones show differences in open chromatin at the *CFTR* locus. Open chromatin mapping of 16HBE14o^−^ WT (black) and the mean of two clones each of CFTR^high^ (clones 4 and 7, brown) and CFTR^low^ (clones 3 and 12, blue) cells, mapped to the hg19 genome build. Clonal cells were collected at two non‐sequential passages. Specific sites where chromatin is more open in CFTR^high^ or CFTR^low^ clones are marked by arrows.

First, we examined open chromatin profiles of cells from the bulk 16HBE14o^−^ population, CFTR^high^ clones, and CFTR^low^ clones using assay for transposase accessible chromatin with high‐throughput sequencing (ATAC‐seq) (Corces et al., 2017). In our earlier work, we identified peaks of open chromatin corresponding to CREs in multiple cell types including 16HBE14o^−^ (Ott et al., 2009; Yang et al., 2016; Zhang et al., 2012). Similar to open chromatin in other airway cells, in addition to the topologically associated domain (TAD) boundaries at −80.1 kb and +48.9 kb, we see key CREs at −44 kb, −35 kb, and +6.8 kb, and a common peak of open chromatin of undefined function in intron 10 (legacy nomenclature intron 10c, RefSeq intron 11 iii [Yin et al., 2022]). Among airway cells, unique to 16HBE14o^−^ cells are open chromatin peaks at −33 kb, −3.4 kb, and in introns 4 and 23 (legacy), though these sites are seen in some other cell types (NandyMazumdar et al., 2020; Ott et al., 2009; Yang et al., 2016). Compared to bulk 16HBE14o^−^ cells, the profiles of CFTR^high^ and CFTR^low^ clonal cells show the same open chromatin peaks across the *CFTR* locus; however, there are notable differences in the normalized relative height of two peaks when comparing CFTR^high^ and CFTR^low^ profiles (Figures 2 and S2). Firstly, the −33 kb ATAC‐seq peak upstream of the *CFTR* promoter has increased accessibility to the transposase in the CFTR^high^ cells compared to CFTR^low^ or 16HBE14o^−^ bulk cells (Figures 2 and S2, brown arrow). The −33 kb element is adjacent to the strong airway‐selective enhancer at −35 kb, and like this CRE and another enhancer at −44 kb, has weak RNAPII enrichment in 16HBE14o^−^ cells (Figure S2). In contrast, this site is not accessible in ATAC‐seq nor is it enriched for RNAPII in Calu3, a lung carcinoma cell line that also expresses *CFTR* (Figure S2), nor primary bronchial epithelial cells (Kerschner et al., 2021). Conversely, another CRE of unknown function, located in intron 10 (i10c) is of higher intensity in CFTR^low^ cells compare to 16HBE14o^−^ or CFTR^high^ cells (Figures 2 and S2, dark blue arrow).

**FIGURE 3:**
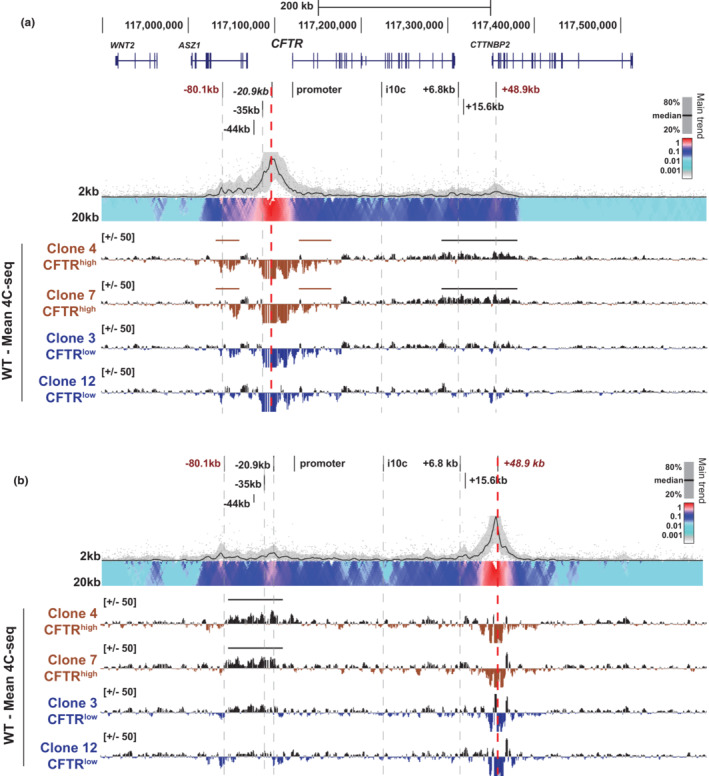
The *CFTR* locus 3D structure in 16HBE14o^−^ cells correlates with *CFTR* expression levels. = 4C‐seq analysis of 16HBE14o^−^ WT (black), CFTR^high^ clones (brown), and CFTR^low^ clones (blue) with viewpoints (red dotted lines) at the −20.9 kb CRE (a) or the +48.9 kb 3’ TAD boundary (b). Key *CFTR* CREs are shown at the top of each panel. For each viewpoint, a 16HBE14o^−^ WT domainogram is shown at the top and below is the subtraction of the read quantification tracks of each CFTR^high^ or CFTR^low^ clone 4C‐seq interaction profile, from 16HBE14o^−^ WT cells in log_2_ scale. Losses and gains in interactions from 16HBE14o^−^ WT are above or below the y‐axis, respectively. Regions of specific interest are marked by horizontal bars.

Next, we investigated whether the 3D organization of the *CFTR* locus was different in CFTR^high^ or CFTR^low^ cells compared to 16HBE14o^−^ cells using circular chromatin conformation capture with deep sequencing (4C‐seq) to measure long‐range chromatin contacts. We used 4C‐seq viewpoints at two key structural elements at the *CFTR* locus that were described previously (NandyMazumdar et al., 2020; Yang et al., 2016): the −20.9 kb CRE, a CCCTC binding factor (CTCF)‐occupied insulator element and the 3′ TAD boundary at +48.9 kb, which also binds CTCF (Blackledge et al., 2007). We first compared the −20.9 kb viewpoint interaction profiles across the *CFTR* locus between 16HBE14o‐ bulk cells and the CFTR^high^ and CFTR^low^ clones (Figure 3a). In the read quantification tracks in Figure 3a, reads above the line in black denote loss of interactions compared to the 16HBE14o‐ bulk cell control interaction profile and reads below the line denote gain in interactions (in brown for CFTR^high^ clones and blue for CFTR^low^ clones). Notably, the CFTR^high^ cells have lower interaction frequencies between the −20.9 kb CRE, downstream enhancer blocking insulator elements at +6.8 kb (which binds CTCF) and +15.6 kb, as well as the 3′ TAD boundary at +48.9 kb compared to 16HBE14o^−^ bulk cells (Figure 3a, black line). This decrease in interactions in CFTR^high^ cells was confirmed reciprocally with a viewpoint at the +48.9 kb 3′ TAD boundary. Compared to bulk 16HBE14o^−^ cells, reduced interactions were seen between this viewpoint, the 5′ TAD boundary at −80.1 kb, upstream enhancers at −44 and −35 kb, as well as the −20.9 kb CRE (Figure 3b, black line). In contrast, there was a slight increase in interactions between the −20.9 kb viewpoint, the *CFTR* promoter and the 5′ end of the coding region in addition to the upstream TAD boundary at −80.1 kb in CFTR^high^ cells compared to 16HBE14o^−^ (Figure 3a, brown lines). There were few changes in interactions with either the −20.9 kb CRE or the +48.9 kb site in CFTR^low^ cells compared to bulk 16HBE14o^−^ cells, (Figure 3a,b). These data suggest that the higher order structure of *CFTR* and intra‐locus interactions correlate with high CFTR expression levels in the CFTR^high^ cells.

### 3.3 The transcriptome of CFTR^high^ and CFTR^low^ cells suggests different cellular identities

**FIGURE 4:**
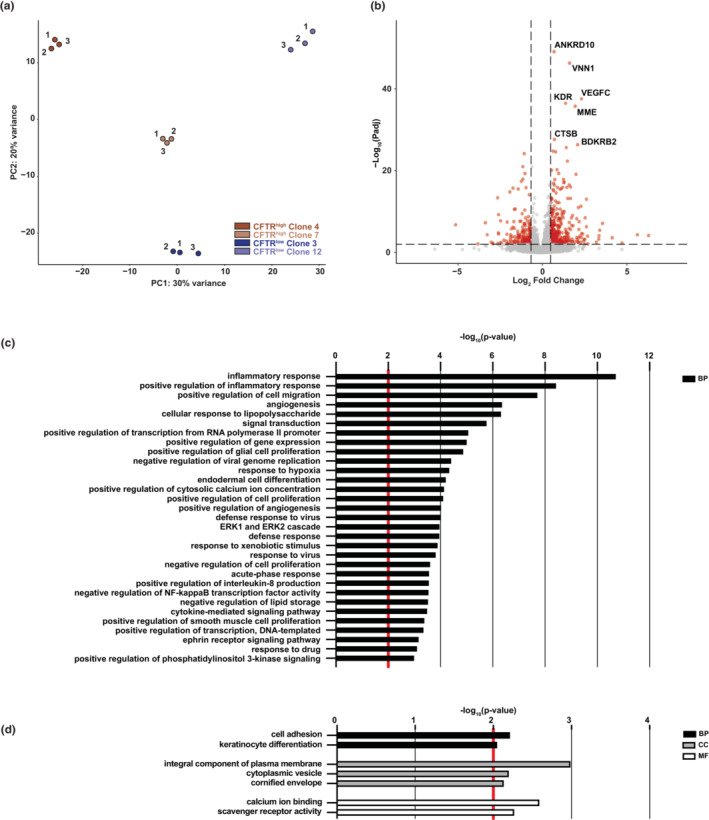
Transcriptomic differences of CFTR^high^ and CFTR^low^ cells. (a) Principal component analysis (PCA) plot comparing RNA‐seq data from individual replicates of CFTR^high^ and CFTR^low^ clones (*n* = 3 of each). (b) Volcano plot of RNA‐seq data comparing CFTR^high^ and CFTR^low^ cells (−log_10_ adjusted *p*‐value vs. log_2_ fold change) with genes upregulated in CFTR^low^ and in CFTR^high^ cells shown to the left and right, respectively. Shown in red are genes with an absolute fold change of ≥1.5 and an adjusted *p*‐value of ≤0.01. (c,d) Gene ontology process enrichment analysis of 311 genes upregulated in CFTR^high^ cells (c) and 169 genes upregulated in CFTR^low^ cells (d), when comparing CFTR^high^ and CFTR^low^. Terms with a *p*‐value of ≤0.01 (denoted by red line) for biological process (BP), cellular component (CC), and molecular function (MF) are shown, with only the top 20 biological process (BP) terms listed in (c).

To determine whether the 16HBE14o^−^ CFTR^high^ and CFTR^low^ cells represented different cell lineages in the bronchial epithelium, we investigated their transcriptome. RNA‐seq was performed in triplicate on the same two clones each of CFTR^high^ and CFTR^low^ cells that were used in the genomic analysis of *CFTR* described above and cells. Principal component analysis (PCA) showed strong correlation between replicates and confirmed clear transcriptomic differences between CFTR^high^ and CFTR^low^ cells (Figure 4a). Differentially expressed genes (DEGs) between the CFTR^high^ (*n* = 6) and CFTR^low^ (*n* = 6) phenotypes were identified by pairwise comparisons, filtering for genes with a fold change ≥1.5 or ≤−1.5, an adjusted *p*‐value ≤0.01, and a basemean >30 (Figure 4b, Table S2). Using these cutoffs, a total of 480 genes were differentially regulated, with 311 genes upregulated in CFTR^high^ cells compared to CFTR^low^ cells and in the reciprocal comparison 169 genes were upregulated in CFTR^low^ cells (i.e., downregulated in CFTR^high^ cells). GO term enrichment was performed on the DEGs upregulated in each set of cells. Among processes upregulated in CFTR^high^ cells, were those associated with the inflammatory response, cellular response to lipopolysaccharide, cell migration, positive regulation of gene expression, the plasma membrane, and extracellular regions (Figure 4c). Genes associated with these terms included cytokines (CXCL6 and CXCL10), interleukins (IL6, IL1B, and IL18R1), integrin alpha 2 (ITGA2), and transcription factors such as ETS homologous factor (EHF), GATA binding protein 6 (GATA6), ETS proto‐oncogene 1, transcription factor (ETS1), and KLF transcription factor 4 (KLF4) (Table S3). Processes upregulated in CFTR^low^ cells included those associated with cell adhesion, the plasma membrane, cytoplasmic vesicle, and calcium ion binding (Figure 4d). Genes included in the cell adhesion biological process include protocadherins (PCDHB2, PCDHB3, PCDHGA2, and PCDHGA4) (Table S4). Protein channels with roles in epithelial cells are encoded by genes such as ATP‐binding cassette subfamily C member 2 (ABCC2), chloride voltage‐gated channel Kb (CLCNKB), transient receptor potential cation channel subfamily V member 6 (TRPV6), and the solute carriers SLC16A14, SLC40A1, and SLC9A4 are included in cellular component terms for the plasma membrane and cytoplasmic vesicle (Table S4). In combination, these transcriptomic profiles suggest that the phenotype of the CFTR^high^ cells is more closely related to secretory epithelial cells in the airway, having functions in the innate immune response and inflammation, while the CFTR^low^ cells do not share a strong identity with any differentiated lung epithelial cell type and are likely adapted to prolonged culture in vitro.

## 4 DISCUSSION

The 16HBE14o^−^ cell line has been widely used as a surrogate for primary HBE cells in many studies of epithelial function in airway disease. However, like other immortalized human cell lines, it has limitations, mainly arising from sequelae of the immortalization procedure and subsequent dedifferentiation in prolonged culture in vitro. Here, we investigated the heterogeneity of the 16HBE14o^−^ cell line, particularly with respect to *CFTR/*CFTR expression since this line and its gene‐edited derivatives are frequently used as the model system of choice in CF research (Bednarski et al., 2016; Erwood et al., 2020; Ko et al., 2022; Michaels et al., 2022; Santos et al., 2022, 2023; Valley et al., 2019). We performed single‐cell cloning to isolate clonal populations of 16HBE14o^−^‐derived cells and found clones that stably express high or low levels of CFTR mRNA and protein. We further characterized two clones each phenotype (Figure 1).

Focusing first on the *CFTR* locus and its CREs, open chromatin profiling revealed accessibility differences at −33 kb upstream of the *CFTR* promoter and at a site within intron 10 (legacy, Refseq intron 11iii) of the gene (Figure 2). The −33 kb CRE, which to date is functionally uncharacterized, is only observed in 16HBE14o^−^ cells and not in the *CFTR*‐expressing lung adenocarcinoma cell line Calu3 (Figure S2) or primary human bronchial epithelial cells (Kerschner et al., 2021; Suzuki et al., 2020). This site is more open in CFTR^high^ cells compared to parental 16HBE14o^−^ or CFTR^low^ cells and it is enriched for RNAPII and H3K27ac in bulk 16HBE14o^−^ cells (NandyMazumdar et al., 2020). Of note, its open chromatin state is not dependent on the presence or position of the strong enhancer at −35 kb in these cells (Kerschner et al., 2022; NandyMazumdar et al., 2020). Inspecting the ENCODE DNaseI hypersensitivity 125 cell types track (Thurman et al., 2012), shows open chromatin at this site only in primary human epidermal keratinocytes from a single donor (Lonza, NHEK), suggesting that this CRE has a highly specialized function in 16HBE14o^−^ cells.

The i10c CRE (Refseq i11iii) was first identified as a site of open chromatin in Caco2 cells, a colorectal adenocarcinoma cell line which expresses *CFTR* (Smith et al., 2000 *)*, however, it lacked enhancer activity in these cells (Phylactides et al., 2002); moreover, it was evident in skin fibroblasts that do not express CFTR (Yang et al., 2016). The i10c CRE chromatin is also open in many cell types profiled in the ENCODE database which do not express *CFTR*. In addition to being a site of open chromatin in 16HBE14o^−^ cells (NandyMazumdar et al., 2020; Zhang et al., 2013), i10c interacts with the transcription factor orthodenticle homeobox 2 (OTX2) where it represses *CFTR* in definitive endoderm cells (Kerschner et al., 2021). Consistent with the earlier data, the accessibility of this element is inversely correlated to *CFTR* expression, again suggesting a repressive role in these cells. Of note, interactions across the *CFTR* locus including the i10c region were diminished in airway cells when *CFTR* expression was de‐repressed (Paranjapye et al., 2022).

While the 3D looping structure of *CFTR* in the CFTR^low^ cells was not substantially different from bulk 16HBE14o^−^ cells, we observed multiple changes to the 3D organization of the locus in CFTR^high^ cells (Figure 3). Many of the same alterations were observed in Calu3 and 16HBE14o^−^ cells in which *CFTR* expression increased following transient depletion or stable deletion of the *CFTR* repressor, Krüppel‐like factor 5 (KLF5) (Paranjapye et al., 2022). In the CFTR^high^ cells, as well as the KLF5‐modulated Calu3 and 16HBE14o^−^ cells, a decrease in the association between the 5′ and 3’ *CFTR* TAD boundaries was observed. Concurrently, increased interactions were detected between the −20.9 kb CTCF‐bound insulator, the *CFTR* promoter and the 5’ TAD boundary. This suggests that in some airway epithelial cells, the unperturbed 3D locus structure supports higher levels of *CFTR* expression.

Examination of the RNA‐seq data comparing CFTR^high^ and CFTR^low^ cells did not reveal DEG profiles that enable clear assignment of a known airway epithelial cell identity (Figure 4). Furthermore, there was no obvious difference in cell morphology when comparing CFTR^high^ and CFTR^low^ cells (data not shown). However, the most significantly upregulated processes: inflammatory response and innate immunity (response to bacteria and viruses) indicate that the CFTR^high^ cells may be more closely related to surface secretory cells in the airway than to other cell types. In addition to experiments on *CFTR* regulation and CFTR function, 16HBE14o^−^ cells are frequently used as a model to study infection and the innate immune response in airway disease. Our data would caution against using clonal isolates of these cells to study inflammation or the immune response, as different responses may reflect the parental cell of origin and not the genetic manipulation under study.

While loss of functional CFTR protein is associated with chronic inflammation in the lung, this is likely due to the recurrent cycles of infection (Cantin et al., 2015; Cohen‐Cymberknoh et al., 2013). None of the cells examined here were exposed to inflammatory agonists during culture; hence, the transcriptomic profiles of the clonal cell lines likely reflect the intrinsic properties of the cell of origin and not merely the levels of *CFTR* protein.

It is unclear from the original reports whether the 16HBE14o^−^ cell line available to researchers today was truly a clonal isolate (Cozens et al., 1994). Irrespective of this, the line has evolved over many decades in culture to become a heterogeneous population of cells. This is not unusual as a consequence of SV40‐mediated immortalization, which may result in loss of allelic heterozygosity and/or increased ploidy (Meisner et al., 1988). In the experiments presented here, we provide evidence for the heterogeneity of the 16HBE14o^−^ cell line when grown as standard 2D cultures. It is possible that further transcriptional divergence may arise if cells are grown under different conditions for example at air–liquid interface on permeable supports, or in response to cellular perturbations or stimuli. It would also be of interest to investigate the proteome of the cells, though in other 16HBE14o^−^ derived cell lines transcriptome and proteome differences were positively correlated (Santos et al., 2023). However, our data encourage caution when interpreting experiments performed in 16HBE14o^−^ cells, and suggest that multiple different clonal populations should be assayed following any genetic manipulation of this line.

## AUTHOR CONTRIBUTIONS

Alekh Paranjapye and Ann Harris conceived the project. Alekh Paranjapye and Jenny L. Kerschner performed experiments. Alekh Paranjapye and Jenny L. Kerschner analyzed data. Alekh Paranjapye, Jenny L. Kerschner, and Ann Harris interpreted results. Jenny L. Kerschner and Ann Harris wrote the manuscript.

## FUNDING INFORMATION

This work was supported by the National Institutes of Health (R01 HL094585 [A.H.]; T32 GM008056 [A. P.]) and the Cystic Fibrosis Foundation (Harris 18P0, Davis 19XX0). The funders had no role in the design of the study, in the collection, analysis, or interpretation of the data; in the writing of the manuscript; or in the decision to publish the results. The content is solely the responsibility of the authors and does not necessarily represent the official views of the funding agencies.

## ETHICS STATEMENT

This study did not involve human or animal subjects.

## DISCLOSURES

The authors declare no conflict of interest.

## Supporting information


Table S1.

Table S2.

Table S3.

Table S4.

## Data Availability

The deep sequencing data supporting the findings of this study are available at GEO accession GSE220855.
