# Supplementary material for: Cellular heterogeneity in the 16HBE14o − airway epithelial line impacts biological readouts
Source: Physiol Rep. 2023 Jun 3;11(11):e15700. doi: 10.14814/phy2.15700 (PMC10238858; doi:10.14814/phy2.15700)
Supplement: Supplementary file 1 — Table S1. [file PHY2-11-e15700-s002.pdf]

Cellular heterogeneity in the 16HBE14o$^-$ airway epithelial line impacts biological readouts

Jenny L. Kerschner, Alekh Paranjapye, and Ann Harris

Data Supplement

Figure Legends

Figure S1: CFTR protein analysis of 16HBE14o$^-$ CFTR^{high} and CFTR^{low} clones.

Figure S2: Open chromatin profiles of individual CFTR^{high} and CFTR^{low} clones.

Table S1: Oligonucleotides

Table S2: DEGs of CFTR-high vs CFTR-low with a fold change ≥ 1.5 or ≤ −1.5, an adjusted p-value ≤ 0.01, and a basemean >30

Table S3: DAVID GO term analysis for CFTR-high DEGs, p-value ≤ 0.01

Table S4: DAVID GO term analysis for CFTR-low DEGs, p-value ≤ 0.01

Supplementary Figure Legends

Figure S1: CFTR analysis of 16HBE14o$^-$ CFTR^{high} and CFTR^{low} clones. Representative western blot showing CFTR protein levels in 16HBE14o$^-$ cells, and 12 clones and β-tubulin loading control with the 4 chosen for subsequent analyses shown in brown (CFTR^{high}) or blue (CFTR^{low}).

Figure S2: Open chromatin profiles of individual CFTR^{high} and CFTR^{low} clones. Open chromatin mapping of 16HBE14o$^-$ CFTR^{high} (clones 4 and 7, brown) and CFTR^{low} (clones 3 and 12, blue) cells, mapped to the hg19 genome build. Clonal cells were collected from two non-sequential passages. Sites where chromatin is more open in CFTR^{high} or CFTR^{low} clones are marked by arrows. At the top for comparison are shown Calu3 ATAC-seq and RNA-polII ChIP-seq (in dark grey) and 16HBE14o$^-$ WT ATAC-seq RNA-polII ChIP-seq (in black) (GSE132808).

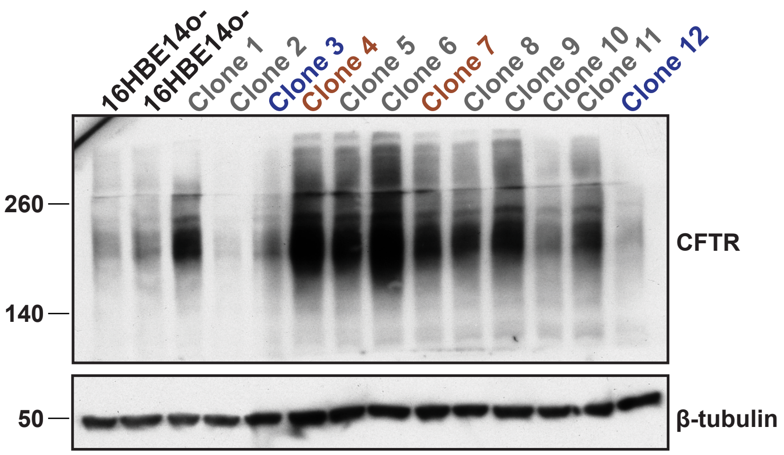

16HBE14o-
16HBE14o-
Clone 1
Clone 2
Clone 3
Clone 4
Clone 5
Clone 6
Clone 7
Clone 8
Clone 9
Clone 10
Clone 11
Clone 12
260
140
CFTR
50
β-tubulin

Figure S1

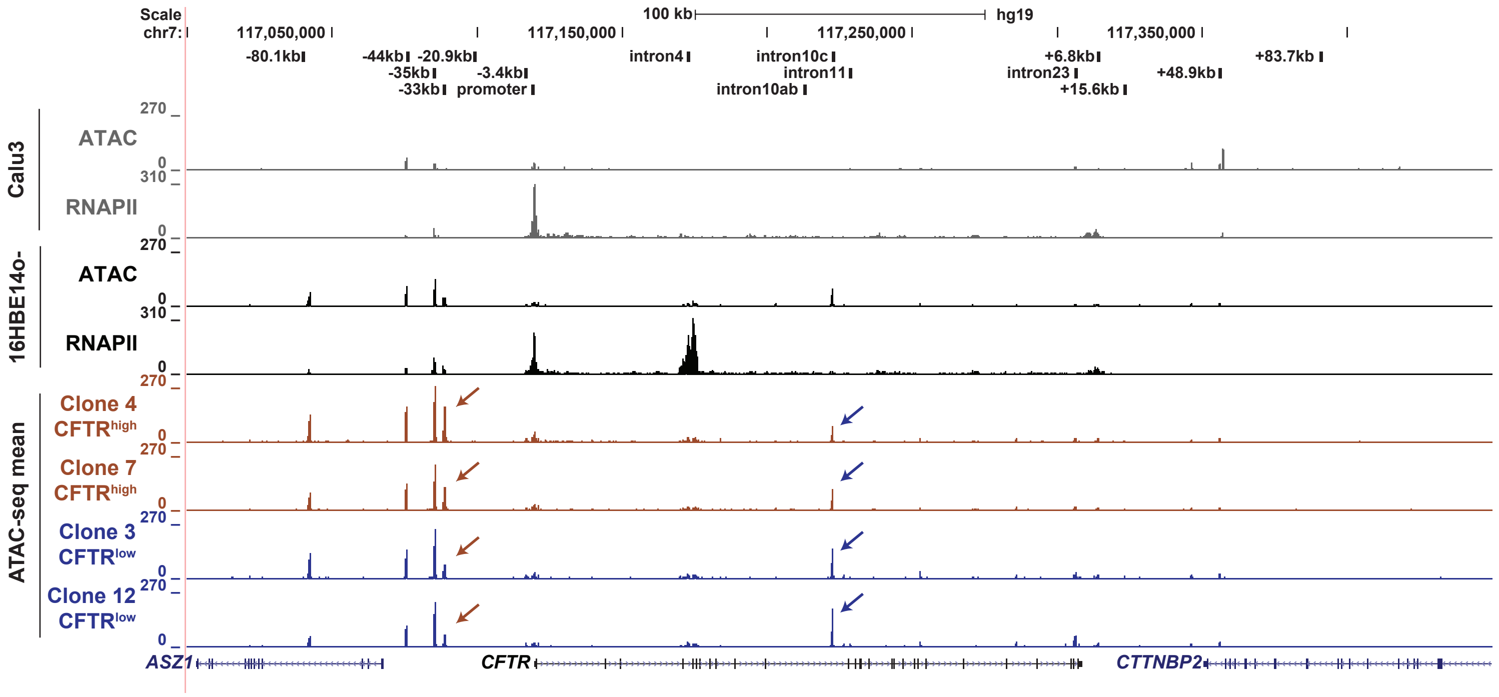
Scale
chr7:
100 kb
hg19
117,050,000
117,150,000
117,250,000
117,350,000
-80.1kb
-44kb
-20.9kb
-35kb
-3.4kb
-33kb
promoter
intron4
intron10c
intron11
intron10ab
+6.8kb
intron23
+15.6kb
+48.9kb
+83.7kb
Calu3
ATAC
RNAPII
16HBE14o-
ATAC
RNAPII
ATAC-seq mean
Clone 4 CFTRhigh
Clone 7 CFTRhigh
Clone 3 CFTRlow
Clone 12 CFTRlow
270
310
0
ASZ1
CFTR
CTTNBP2

Figure S2

Table S1: Oligonucleotides

TaqMan Assay

Gene	Sequence (5'>3')
CFTR	AGCTGTCAAGCCGTGTTCTAGATA
	ATGAGGAGTGCCACTTGCAAA
	/56-FAM/CACACGAAA/ZEN/TGTGCCAATGCAAGTCCTT/3IABkFQ/
B2M	AAGTGGGATCGAGACATGTAAG
	GCAAGCAAGCAGAATTTGGA
	/56-JOEN/TCATGGAGG/ZEN/TTTGAAGATGCCGCA/3IABkFQ/

4C-seq

Viewpoint (enzyme combo.)	Reading Primer (5'>3')	Non-reading Primer (5'>3')
DHS -20.9 kb (NlaIII/DpnII)	tacacgacgctcttccgatctTTAACAAAGTTTAGGTAAATGACCA	actggagttcagacgtgtgctcttccgatctCAAAGTGAGCTATTTTGTTTTCTC
DHS +48.9 kb (NlaIII/DpnII)	tacacgacgctcttccgatctTACACGACGCTCTTCCGATCT	actggagttcagacgtgtgctcttccgatctGAGTGAGCTTGAAAGCCATG
